# *Streptococcus agalactiae* Causing Neonatal Infections in Portugal (2005–2015): Diversification and Emergence of a CC17/PI-2b Multidrug Resistant Sublineage

**DOI:** 10.3389/fmicb.2017.00499

**Published:** 2017-03-28

**Authors:** Elisabete R. Martins, Cristiano Pedroso-Roussado, José Melo-Cristino, Mário Ramirez, The Portuguese Group for the Study of Streptococcal Infections, Henrique Oliveira

**Affiliations:** Faculdade de Medicina, Instituto de Microbiologia, Instituto de Medicina Molecular, Universidade de LisboaLisbon, Portugal

**Keywords:** *Streptococcus agalactiae*, invasive disease, neonates, CC17, antimicrobial resistance

## Abstract

The molecular characterization of 218 GBS isolates recovered from neonatal invasive infections in Portugal in 2005–2015 revealed the existence of a small number of genetically distinct lineages that were present over a significant time-span. Serotypes III and Ia were dominant in the population, together accounting for >80% of the isolates. Clonal complex 17 included 50% of all isolates, highlighting the importance of the hypervirulent genetic lineage represented by serotype III ST17/*rib*/PI-1+PI-2b. Serotype Ia was represented mainly by ST23, previously reported as dominant among invasive disease in non-pregnant adults in Portugal, but also by ST24, showing an increased frequency among late-onset disease. Overall erythromycin resistance was 16%, increasing during the study period (*p* < 0.001). Macrolide resistance was overrepresented among CC1 and CC19 isolates (*p* < 0.001 and *p* = 0.008, respectively). While representatives of the hypervirulent CC17 lineage were mostly susceptible to macrolides, we identified for the first time in Europe a recently emerging sublineage characterized by the loss of PI-1 (CC17/PI-2b), simultaneously resistant to macrolides, lincosamides, and tetracycline, also exhibiting high-level resistance to streptomycin and kanamycin. The stability and dominance of CC17 among neonatal invasive infections in the past decades indicates that it is extremely well adapted to its niche; however emerging resistance in this genetic background may have significant implications for the prevention and management of GBS disease.

## Introduction

*Streptococcus agalactiae*, or group B streptococci (GBS), is a leading cause of severe neonatal infections (Edmond et al., 2012), while also being a frequent colonizer of the genitourinary and gastrointestinal tracks of a significant proportion of the human population. The onset of GBS infections takes place early in infancy, usually within the first week of life, being designated early-onset disease (EOD). Late-onset disease (LOD) develops after the first week until 3 months of age. Maternal colonization with GBS is the leading risk factor for the development of neonatal disease, but other sources of transmission exist (Manning et al., 2004).

Even though national health authorities formally implemented universal GBS screening of pregnant women only in 2013 (Norma 37/2011, 2013), the neonatology section of the Portuguese Society of Paediatrics issued guidelines for screening and prevention of GBS perinatal disease in 2004. These recommended universal screening of pregnant women at 35–37 weeks gestation and the administration of intrapartum antimicrobial prophylaxis (IAP) to GBS carriers (Pinheiro et al., 2013). Following the publication of these guidelines, a study based on voluntary reporting of GBS neonatal disease in Portugal described reductions in the incidence of EOD of nearly 40%, as well as of the case-fatality rate associated (Neto, 2008), in agreement with what had been observed in other countries where similar measures were implemented (Verani et al., 2010).

Penicillin is the first line agent for prevention and treatment of GBS disease, while clindamycin and vancomycin are considered suitable alternatives for penicillin-allergic patients (Verani et al., 2010). GBS is mostly considered uniformly susceptible to β-lactams. In contrast, increasing resistance to erythromycin and clindamycin in both neonatal and adult invasive infections have been reported worldwide in recent years (Castor et al., 2008; Lamagni et al., 2013), raising concern as to the long-term efficacy of current prophylatic and therapeutic strategies. Development of a universal vaccine is the most promising approach to fight GBS disease, given the potential adverse effects of IAP and the need for effective prevention of both neonatal and adult infections. In recent years, capsular polysaccharide conjugated vaccines have reached phase II clinical trials while a pilus-based vaccine is also being investigated (Nuccitelli et al., 2015).

Serotyping is the classical phenotypic typing method for the characterization of GBS, allowing their classification into capsular polysaccharide-based serotypes Ia to IX. More recently, genotypic tools such as multilocus sequence typing (MLST) have contributed to the recognition of particular genetic lineages within serotypes that were shown to differ in virulence potential and tropism (Jones et al., 2003). The combination of several typing methods, including surface protein gene (Creti et al., 2004) and pilus-islands (Martins et al., 2013) profiling were shown to afford increased discriminatory power. Currently, a genetic lineage of serotype III defined by sequence type 17 (ST17), surface protein *rib* and the combination of pilus islands 1 and 2b is disseminated worldwide and is associated with an increased ability to cause neonatal invasive disease, particularly meningitis (Manning et al., 2009; Meehan et al., 2014). On the other hand, invasive disease among adults has been mostly attributed to the serotype Ia/ST23/*eps* and serotype V/ST1/*alp3* clones (Lamagni et al., 2013; Meehan et al., 2014; Teatero et al., 2014).

The aim of our study was to perform the characterization of a collection of GBS isolates recovered from neonates with invasive disease in Portugal in 2005–2015, to document the prevalence of serotypes, genetic lineages and antimicrobial resistance patterns.

## Materials and methods

### Bacterial isolates

This work was part of a laboratory-based surveillance program in which microbiology laboratories of 22 Portuguese hospitals or hospital centers were asked to submit to a central laboratory all GBS isolates recovered from cases of neonatal GBS invasive disease.

Cases were classified into three presentations: EOD (early-onset disease), defined as disease onset from birth to 6 days after birth; LOD (late-onset disease), for patients ranging from 7 to 90 days of age; and ULOD (ultra-late-onset disease), in which the patients were older than 91 days of life but <1 year. Whenever, GBS isolates were available from more than one sample from the same patient, only the first isolate was included in the study. Identification was confirmed to the species level by Gram stain, colony morphology, and a commercial latex agglutination technique (Slidex Strepto B; bioMérieux, Marcy L'Étoile, France).

### Serotyping

Capsular serotyping was performed by a slide agglutination assay with IMMULEX™ STREP-B Kit (Statens Serum Institute, Copenhagen, Denmark) according to the manufacturer's instructions.

### Antimicrobial susceptibility testing

Susceptibility testing was performed by disc diffusion according to the Clinical and Laboratory Standards Institute (CLSI) methods and interpretation criteria for *Streptococcus* spp. β-Hemolytic Group (Clinical Laboratory Standards Institute, 2014). The panel of antibiotics included penicillin G, erythromycin, clindamycin, vancomycin, chloramphenicol, levofloxacin, and tetracycline. We have also performed a screening test by disk diffusion for detection of High-Level Aminoglycoside Resistance (HLAR), with 120 μg gentamicin and 300 μg streptomycin disks, according to the CLSI methods and interpretative criteria for *Enterococcus* species (Clinical Laboratory Standards Institute, 2014). Susceptibility testing of kanamycin and lincomycin was performed as recommended by the Société Française de Microbiologie (http://www.sfm-microbiologie.org).

Resistance to macrolides only (M phenotype); and constitutive or inducible cross-resistance to macrolides, lincosamides and streptogramin B (cMLS_B_ and iMLS_B_ phenotypes, respectively), were determined by a double-disk test with erythromycin and clindamycin. The presence of macrolide resistance genes was detected by multiplex PCR targetting the *erm*(B), *erm*(A) [*erm*(TR) subclass], and *mef* [*mef* (A) or *mef* (E)] genes (Martins et al., 2012), and by an additional PCR for the *erm*(T) gene (Compain et al., 2014). The presence of lincosamide resistance genes *lsa*(C) (Malbruny et al., 2011) and *lnu*(B) (Bozdogan et al., 1999) was also tested by PCR.

All tetracycline-resistant isolates were screened for the presence of the *tet*(K), *tet*(L), *tet*(M), and *tet*(O) genes, as previously described (Martins et al., 2012). The genetic determinants of High-Level Resistance (HLR) to aminoglycosides *aac*(*6*′)-*aph*(*2*″), *aph*(*2*″)-*Ib, aph*(*2*″)-*Ic, aph*(*2*″)-*Id, aph*(*3*′)-*III, ant*(*4*′)-*Ia*, and *ant*(*6*)-*Ia* were tested by PCR as described for enterococci (Clark et al., 1999; Vakulenko et al., 2003).

### MLST

MLST was performed as described previously (Jones et al., 2003) and sequence type (ST) assignment was done according to the *S. agalactiae* MLST database (http://pubmlst.org/sagalactiae). Analysis of DNA sequences was performed by using the Bionumerics software (Applied Maths, Sint-Martens-Latem, Belgium). Alleles and sequence types not previously described were deposited in the *S. agalactiae* MLST database. The goeBURST algorithm implemented in PHYLOViZ software (Nascimento et al., 2017) was used to establish relationships between STs. Clonal complexes (CCs) were defined at the single-locus-variant (SLV) level.

### Surface protein gene profile and Pili

The GBS alpha (*bca*) and alpha-like (*eps, rib, alp*2/3, and *alp*4) protein genes were detected by a multiplex PCR assay (Creti et al., 2004), and the *alp*2 and *alp*3 genes differentiated as previously described (Martins et al., 2010).

All isolates were tested for the presence of PI-1, PI-2a, and PI-2b loci by PCR (Martins et al., 2013).

### Typing analysis and statistics

Information about the number of live births in Portugal was obtained from Statistics Portugal (http://www.ine.pt).

Simpson's index of diversity (SID) and 95% confidence intervals (CI_95%_) was used to estimate the diversity found among the isolates studied (www.comparingpartitions.info; Carrico et al., 2006).

Differences were evaluated by the Fisher exact test with the false discovery rate (FDR) correction for multiple testing (Benjamini and Hochberg, 1995). The Cochran-Armitage test was used for trends. A *p* < 0.05 was considered significant for all tests.

### Ethics statement

Case reporting and isolate collection were considered surveillance activities and were exempt from evaluation by the Review Board of the Faculdade de Medicina da Universidade de Lisboa. The data and isolates were de-identified so that these were irretrievably unlinked to an identifiable person.

## Results

### Isolates

**Figure 1:**
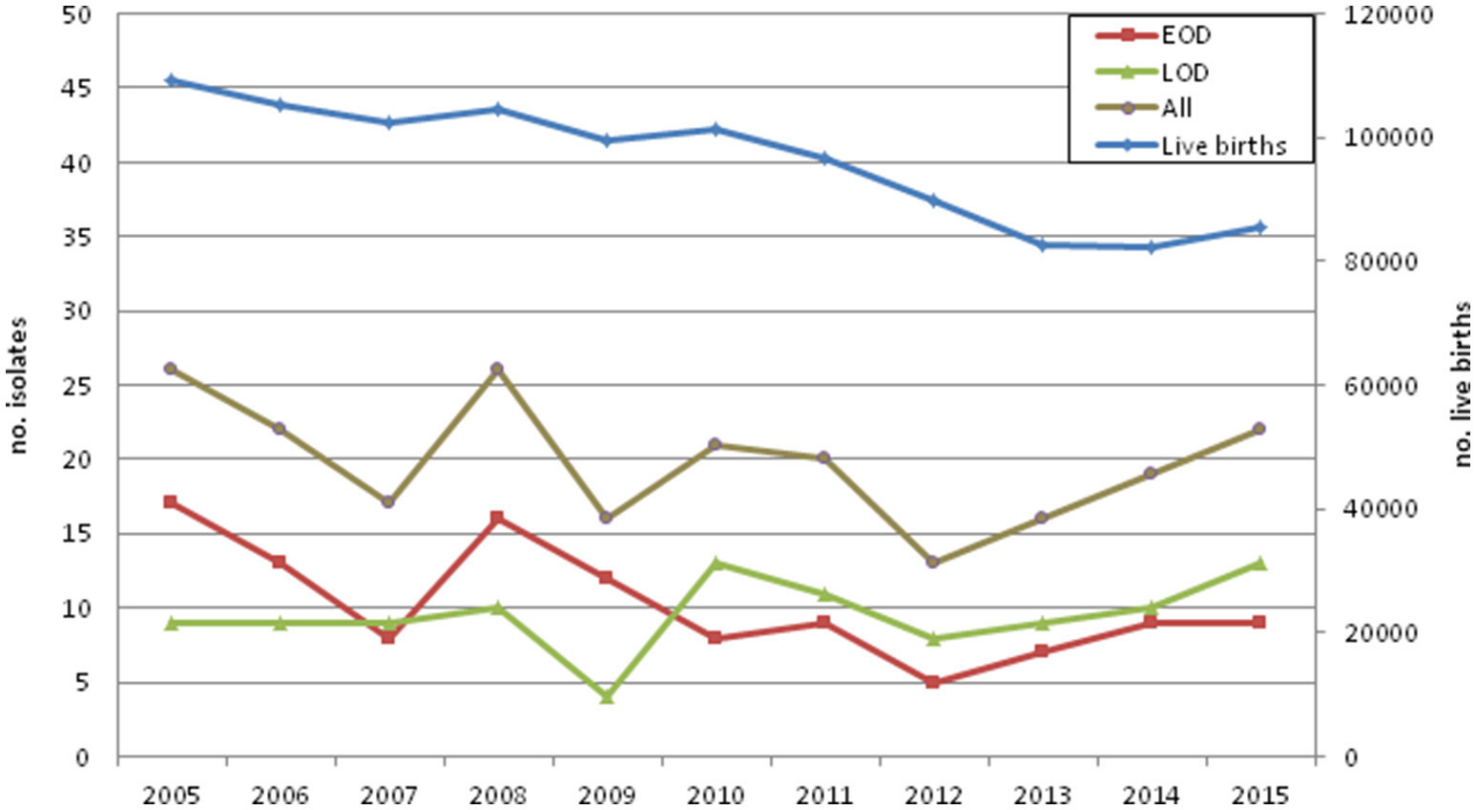
**Invasive neonatal disease cases and live births in Portugal by year**.

Between 2005 and 2015, 218 cases of neonatal invasive group B streptococcal infections were identified, including 188 isolates recovered from blood, 26 from cerebrospinal fluid, 3 from synovial fluid, and one from ascitic fluid. There was a higher number of EOD cases (*n* = 113), followed by LOD (*n* = 101) and 4 ULOD cases (Table 1). The number of births in Portugal declined during the study period and there were yearly variations in disease cases (Figure 1, Supplementary Table 1). For the purpose of statistical analysis ULOD cases were included in LOD.

**Table 1:** **Serotype distribution according to disease onset**.

**Serotype**	**Disease onset, no. (%)^a^**			**Total no (%)**
	**EOD**	**LOD**	**ULOD**	
Ia	26 (11.9)	22 (10.1)	0	48 (22.0)
Ib	8 (3.7)	3 (1.4)	1 (0.5)	12 (5.5)
II	9 (4.1)	0	0	9 (4.1)
III	59 (27.1)	67 (30.7)	2 (0.9)	128 (58.7)
IV	0	3 (1.4)	0	3 (1.4)
V	4 (1.8)	5 (2.3)	0	9 (4.1)
VI	1 (0.5)	0	0	1 (0.5)
VIII	1 (0.5)	0	0	1 (0.5)
IX	1 (0.5)	0	0	1 (0.5)
NT^b^	4 (1.8)	1 (0.5)	1 (0.5)	6 (2.8)
Total	113 (51.8)	101 (46.3)	4 (1.8)	218 (100.0)

a *EOD, early-onset disease; LOD, late-onset disease; ULOD, ultra-late-onset disease*.

b *NT, non-typeable*.

### Serotype distribution

Serotyping results are summarized in Table 1. Overall serotypes were similarly distributed among EOD and LOD (*p* = 0.063), except serotype II that was exclusively represented in EOD, and the three serotype IV isolates that were recovered from LOD cases (Table 1). No statistically significant associations were found between serotype and isolate source. Serotypes III (*n* = 128; 59%) and Ia (*n* = 48; 22%) were the most frequent in the population, together accounting for 81% of the isolates. In spite of almost all known GBS serotypes having been found, including serotypes VIII and IX here described for the first time in Portugal, diversity was low (*SID* = 0.602, CI_95%_ 0.540–0.665).

### Genetic lineages

According to MLST, the 218 isolates presented a high genetic diversity, being distributed across 35 sequence types (*SID* = 0.789, CI_95%_ 0.738–0.839), of which six (ST742–745, ST757, and ST878) were newly identified in this study. The STs clustered into 7 clonal complexes. The distribution of STs, serotypes, surface protein and pilus genes, as well as antimicrobial resistance genotypes across CCs is shown in Table 2.

**Table 2:** **Distribution of serotypes, surface protein, pili and antimicrobial resistance profiles among MLST-based clonal complexes**.

**Genetic lineage^a^**	**Serotype^b^ (*n*)**	**Alp gene^c^**	**Pilus island**	**Macrolide resistance phenotype^d^ (*n*)**	**Antimicrobial resistance genotype^e^ (n)**
**CC1 (*n* = 21)**					
ST1	Ib (7)	*alp3*	PI-1+PI-2a	cMLS_B_(6)	*ermB*(6), *tetM*(7)
	V (6)	*alp3*	PI-1+PI-2a	cMLS_B_(3)	*ermB*(2), *ermTR*(1), *tetM*(2)
	VI (1)	*bca*	PI-1+PI-2a		
ST2	V (2)	*eps*	PI-1+PI-2a		*tetM*(1)
	Ib (1)	*eps*	PI-2a		
	NT (1)	*eps*	PI-2a		*tetM*(1)
ST196	IV (1)	*eps*	PI-1+PI-2a		*tetM*(1)
	Ia (1)	*eps*	PI-1+PI-2a		*tetM*(1)
ST878	NT (1)	*alp3*	PI-1+PI-2a	cMLS_B_	*ermB, tetM*(1)
**CC10 (*n* = 9)**					
ST10	Ib (1)	*eps*	PI-1+PI-2a		*tetM*(1)
	NT (2)	*eps*	PI-1+PI-2a		
ST8	Ib (1)	*bca*	PI-1+PI-2a		*tetM*(1)
ST9	Ib (1)	*bca*	PI-1+PI-2a		
ST12	II (2)	*bca*	PI-1+PI-2a		*tetM*(1), *tetO*(1)
	Ib (1)	*bca*	PI-1+PI-2a	cMLS_B_	*ermB, tetM*(1)
	V (1)	*eps*	PI-1+PI-2a		*tetM*(1)
**CC17 (*n* = 109)**					
ST17	III (93)	*rib*	PI-1+PI-2b	cMLS_B_(2), M(1)	*ermB*(2), *mefE*(1), *tetM*(70), *tetM*+*tetL*(3), *tetM*+*tetO*(2), *aac(6′)-aph(2″)* (1)
	III (8)	*rib*	PI-2b	cMLS_B_(7)	*ermB*(7), *tetM*+*tetO*(4), *tetO*(3), *aph*(*3′*)-*III*+*ant(6)-Ia*(6)
ST757	III (1)	*rib*	PI-2b	cMLS_B_(1)	*ermB, tetM*+*tetO, aph*(*3′*)-*III*+*ant(6)-Ia*(1)
ST109	III (6)	*rib*	PI-1+PI-2b		*tetM*(1)
ST147	III (1)	*rib*	PI-1+PI-2b		*tetM*(1)
ST287	III (2)	*rib*	PI-1+PI-2b		*tetM*(1)
ST290	III (1)	*rib*	PI-1+PI-2b		*tetM*(1)
ST450	III (1)	*rib*	PI-1+PI-2b		*tetM*(1)
ST482	III (1)	*rib*	PI-1+PI-2b		*tetM*+*tetL*(1)
ST550	III (1)	*rib*	PI-1+PI-2b		*tetM*(1)
ST743	III (1)	*rib*	PI-1+PI-2b		*tetM*(1)
ST744	III (1)	*rib*	PI-1+PI-2b		*tetM*(1)
**CC19 (*n* = 25)**					
ST19	III (14)	*rib*	PI-1+PI-2a	cMLS_B_(2), iMLS_B_(5)	*ermB*(1), *ermB*+*lsa*C(1), *ermTR*(5), *tetM*(10)
ST27	III (1)	*rib*	PI-1+PI-2a		*tetM*+*tetO*(1)
ST28	II (5)	*rib*	PI-1+PI-2a		*tetM*(5)
	VIII (1)	*rib*	PI-1+PI-2a		
ST182	III (1)	*rib*	PI-1+PI-2a	cMLS_B_	*ermB, tetO*(1)
ST335	III (1)	*rib*	PI-1+PI-2a	iMLS_B_	*ermTR, tetM*(1)
ST510	III (1)	*rib*	PI-1+PI-2a		*tetM*(1)
ST742	III (1)	*rib*	PI-1+PI-2a		*tetM*(1)
**CC22 (*n* = 3)**					
ST22	II (2) NT (1)	*bca bca*	PI-1+PI-2a PI-1+PI-2a	cMLS_B_(1)	*ermB*(1), *tetM*(1) *tetM*(1)
**CC23 (*n* = 50)**					
ST23	Ia (29)	*eps*	PI-2a	M(3)	*mefE*(3), *tetM*(29) *tetM*+*tetO*(1)
	NT (1)	*bca*	PI-1+PI-2a		
ST88	Ia (1)	*alp2*	PI-1+PI-2a		
ST144	Ia (1)	*rib*	PI-2a		*tetM*(1)
ST745	Ia (1)	*eps*	PI-2a		*tetM*(1)
ST24	Ia (9)	*bca*	PI-2a		*tetM*(9)
ST452	IV (2)	*bca*	PI-1+PI-2a		
ST498	Ia (6)	*bca*	PI-2a		*tetM*(6)
**CC130 (*n* = 1)**					
ST130	IX (1)	*bca*	PI-2a		

a *CC, clonal complex; ST, sequence type*.

b *NT, non-typeable*.

c *Alp, alpha/alpha-like protein*.

d *M, resistance to macrolides; MLS_B_, resistance to macrolides, lincosamides and streptogramins B. The prefix letter refers to the constitutive expression of this phenotype (cMLS_B_) or inducible expression of the phenotype (iMLS_B_)*.

e *All different combinations of antimicrobial resistance genes are presented independently. The erm and mef genes are responsible for the MLS_B_ and M phenotypes respectively while the tet genes confer resistance to tetracycline*.

Half of the collection was represented by CC17 (*n* = 109; 50%), including the serotype III hypervirulent lineage defined by ST17 and its SLVs, surface protein gene *rib* and the combination of PI-1 and PI-2b. CC17 was overrepresented in LOD cases (*p* = 0.007), in agreement with our previous findings and those reported elsewhere (Manning et al., 2009; Martins et al., 2011; Meehan et al., 2014).

Serotype Ia was found in CC23, including mostly ST23/*eps*/PI-2a, but also a sublineage constituted by a double-locus variant of ST23 represented by ST24/*bca*/PI-2a, which has been mostly detected in the Mediterranean region (Martins et al., 2011). ST23 and ST24 and corresponding SLVs were largely distinguished according to disease onset: while ST23 and SLVs (ST88, ST144, and ST745) were dominant among EOD cases (*n* = 23/26, 88.5%), 14 out of the 17 isolates (82.3%) represented by ST24 and SLVs (ST452 and ST498) were recovered from LOD cases (*p* < 0.001).

A small number of isolates was represented by ST1 and the combination of surface protein gene *alp3* and pilus islands 1 and 2a, a genetic background that is usually associated with serotype V. However, out of the 13 isolates identified as ST1/*alp3*/PI-1+PI-2a, 7 presented serotype Ib and only 6 the expected serotype V.

### Antimicrobial susceptibility testing

All isolates were susceptible to penicillin, vancomycin and levofloxacin. Gentamicin, chloramphenicol and streptomycin resistance was found in 0.5% (*n* = 1), 1.4% (*n* = 3), and 3.2% (*n* = 7) of the isolates, respectively.

**Figure 2:**
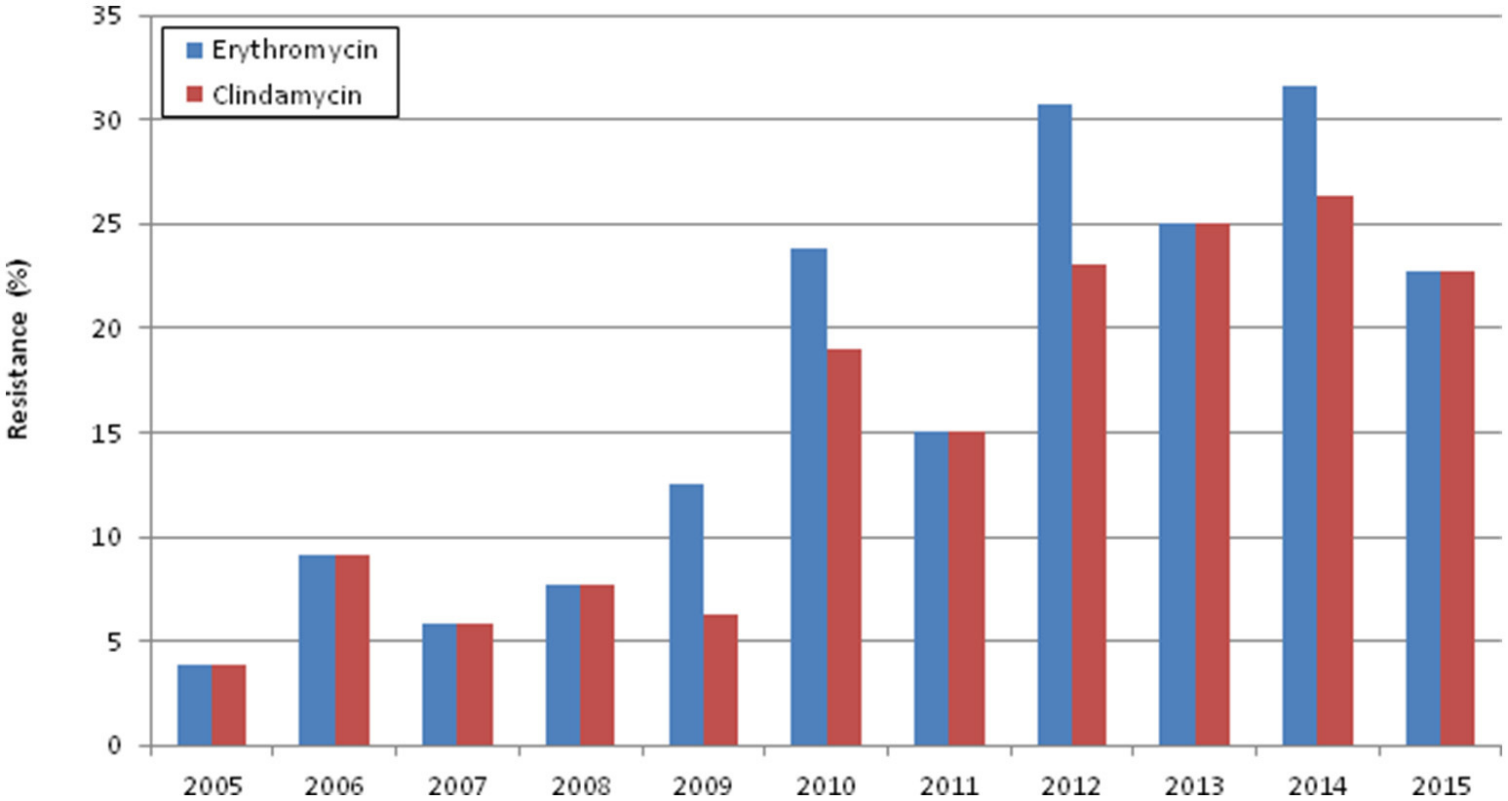
**Erythromycin and clindamycin resistance in the study period**.

The overall rate of erythromycin resistance was 16.1% (*n* = 35) and of clindamycin 14.2% (*n* = 31). Macrolide resistance increased throughout the study period (Cochran-Armitage test of trend, *p* < 0.001) (Figure 2), mainly represented by the cMLS_B_ phenotype (*n* = 25), followed by iMLS_B_ (*n* = 6) and M (*n* = 4) phenotypes. Even though no isolates resistant only to clindamycin were detected, one isolate presenting the cMLS_B_ phenotype carried both the *erm*(B) and *lsa*(C) genes (Table 2), while the *lnu*(B) gene was not found in any isolate. Macrolide resistance was significantly associated with CC1 and CC19 (*p* < 0.001 and *p* = 0.008, respectively), contrasting with CC17 and CC23 in which it was underrepresented (both cases, *p* = 0.03). Interestingly, within CC17 there was a subset of isolates carrying only PI-2b in which macrolide resistance was overrepresented (*p* < 0.001). Most of these isolates also presented HLR to streptomycin, harboring two genetic determinants encoding aminoglycoside-modifying enzymes, *aph(3*′*)-III* and *ant*(*6*)-*Ia* (Table 2). Additional antimicrobial susceptibility testing performed on this subset revealed that all isolates were also resistant to kanamycin and lincomycin.

The single gentamicin resistant isolate was also found within CC17, (Table 2), carrying the *aac(6*′*)-aph(2*″*)* gene, which encodes a bifunctional aminoglycoside-modifying enzyme known to mediate HLR to virtually all clinically available aminoglycosides, but not to streptomycin (Vakulenko et al., 2003).

A high percentage of isolates was resistant to tetracycline (*n* = 187; 85.8%). The resistance determinant most frequently found was *tet*(M) (*n* = 182/187; 97.3%), but different combinations of *tet* genes were also found in a small fraction of the isolates. Within the abovementioned subset of CC17 presenting only PI-2b, all isolates carried the *tet*(O) gene (Table 2).

## Discussion

This study presents the characterization of GBS recovered from invasive infections among neonates in Portugal in 2005–2015. Even though our network comprised most of the hospital microbiology laboratories in Portugal, this study was based on voluntary reporting and is therefore not population based. Notwithstanding, our surveillance network is stable and we consider that any differences observed reflect true variations in cases, still given potentially incomplete reporting we refrained from calculating incidences of GBS disease in Portugal.

No information about the implementation and effectiveness of screening recommendations and IAP are available in Portugal from official sources. When these measures started being implemented at hospital level in Portugal, a decreasing incidence of neonatal GBS disease in 2002–2004 was found, particularly among EOD cases, suggesting that the recent implementation of IAP was already having a beneficial impact (Neto, 2008). In contrast, when comparing the number of neonatal infections with the number of live births in Portugal in 2005–2015 (Figure 1), we observed significant fluctuations in the number of disease cases in the first years of the study, with a potential increase in infections taking place after 2011 (Figure 1). While this increment did not reach statistical significance (*p* = 0.08), our observations support the notion that the implementation of universal screening and IAP may have had a limited impact, and is not currently causing further decreases in neonatal invasive infections in Portugal. In agreement with our findings, in England and Wales (Lamagni et al., 2013), the Netherlands (Bekker et al., 2014), and Iceland (Oladottir et al., 2011), neonatal invasive disease has been shown to increase over time in spite of the implementation of obstetric risk-based recommendations for the prevention of GBS disease.

In a previous study in which we compared the GBS populations associated with vaginal carriage in pregnant women and invasive neonatal infections in Portugal, serotypes Ia and III amounted to 69% of all invasive isolates, and were statistically associated with EOD and LOD, respectively (Martins et al., 2007). In the same study, an overrepresentation of serotype III was found in isolates recovered from the CSF, particularly among LOD cases (Martins et al., 2007), in agreement with other reports suggesting an association of this serotype with meningitis (Manning et al., 2009). In this collection, serotypes III and Ia were even more dominant, together accounting for 81% of all isolates, similarly to what has been reported recently worldwide (Ferrieri et al., 2013; Lamagni et al., 2013; Meehan et al., 2014; Teatero et al., 2014). Even though serotype III was the most frequent among meningitis cases (*n* = 20/26; 77%), this association was not statistically significant.

Only four isolates did not cluster in any of the five most frequent CCs, together responsible for the majority of invasive infections in humans: CC1, CC10, CC17, CC19, and CC23 (Bekker et al., 2014; Meehan et al., 2014; Teatero et al., 2014). We found no significant differences in the relative frequency of CCs over time, indicating that the genetic lineages responsible for neonatal infections in Portugal have been dominant over a significant time-span. Half of our collection was part of the CC17 hypervirulent lineage, sharing the same type III capsular polysaccharide, the surface protein gene *rib* and the combination of PI-1 and PI-2b, with the exception of a small subset of theses isolates carrying only PI-2b, a finding not previously observed in other CC17 isolates from Portugal (Martins et al., 2013). A recent study from Canada, using whole genome sequencing analysis, revealed the loss of PI-1 in a group of CC17 isolates and integration in the same genomic location of a mobile genetic element carrying multiple antimicrobial resistance genes (Teatero et al., 2016). Another recent publication from China confirmed and extended these findings, further discriminating the CC17/PI-2b isolates into two groups, distinguishable by their antimicrobial resistance profiles, namely lincomycin resistance associated with the presence of *lnu*(B) gene (Campisi et al., 2016). In our subset of CC17/PI-2b, all but two isolates were simultaneously resistant to erythromycin, clindamycin, tetracycline and streptomycin, and carried the *erm*(*B*), *tet*(*O*), *aph*(*3*′)-*II, and ant(6)-Ia* genes (Table 2). We found that our CC17/PI-2b also presented resistance to kanamycin and lincomycin, which would place them in the lincomycin resistant sublineage according to Campisi (Campisi et al., 2016). However, none of the our isolates harbored the *lnu*(B) gene, raising the possibility that our strains represent yet another sublineage of CC17/PI-2b.

Taken together with the fact that all our CC17/PI-2b isolates were recovered after 2010, our data suggests that highly resistant sublineages emerged recently and persist within the hypervirulent clone. To our knowledge this is the first report of a CC17/PI-2b lineage in Europe. However, considering that aminoglycoside susceptibility testing is not routinely performed, and given the lack of specific guidelines for streptococci, these highly resistant isolates may be expanding unnoticed in other countries.

Serotype Ia was represented by two sublineages within CC23: ST23/*eps*/PI-2a and its DLV ST24/*bca*/PI-2a. While ST23 and SLVs were dominant among EOD, ST24 and SLVs were associated with LOD cases (*p* < 0.001), indicating that within the same clonal complex, particular sublineages may be better adapted to cause specific disease presentations. Furthermore, the proportion of ST24 and SLVs within CC23 appears to be increasing when compared to our previous studies. While in this collection the ratio ST24/CC23 was 34% (*n* = 17/50), in a previous study including both invasive, non-invasive and colonization strains it was 26% (*n* = 18/69) (Martins et al., 2013). Taken together, these data suggest that not only is ST24 well established in the Mediterranean region, but its frequency may be increasing together with a higher propensity to cause LOD.

A small number of isolates was represented by ST1/*alp3*/PI-1+PI-2a. This genetic lineage is dominant among serotype V isolates and has been mostly found among invasive disease cases in non-pregnant adults (Martins et al., 2012; Meehan et al., 2014). An interesting finding in our study was a subset of serotype Ib isolates identified as ST1/*alp3*/PI-1+PI-2a. Considering that we have not found other serotype Ib isolates with these characteristics in any of our previous studies, as well as in the literature, it is likely that these isolates are the result of a recent capsular switching event (Martins et al., 2010).

Penicillin and ampicillin are first line drugs for prevention and treatment of GBS disease. Penicillin-allergic patients should be given clindamycin, and vancomycin is the option for clindamycin-resistant isolates (Verani et al., 2010). In our study, macrolide and lincosamide resistance rates increased significantly over time (*p* < 0.001) (Figure 2), similarly to what has been reported elsewhere (Castor et al., 2008; Lamagni et al., 2013). Even though overall macrolide resistance among neonates (16.1%) was higher than previously documented among non-pregnant adults (12.9%) in Portugal (Martins et al., 2012), these difference hide fluctuations in time. In fact, in the years common to both studies (2005–2008), macrolide resistance among neonates ranged between 3.9 and 9.1% (Figure 2) whereas among adults it ranged between 14.0 and 20.5% (Martins et al., 2012). These data show that in the same period, macrolide resistance among non-pregnant adults was much higher than in neonates and that resistance in the neonatal isolates has increased significantly only in recent years. Furthermore, in the present study, the cMLSB phenotype was overrepresented and associated with the erm(B) gene, in contrast to that observed previously among non-pregnant adults, in which macrolide resistance was evenly distributed among the cMLSB and iMLSB phenotypes, with the *erm*(A) [*erm*(TR) subclass] gene being the most frequent (Martins et al., 2012). In both studies resistance was mostly found within the same genetic background, represented by ST1/*alp3*/PI-1+PI-2a. However, this lineage expressed serotype V in non-pregnant adults, but in this study presented both serotypes V and Ib, raising the possibility that antimicrobial use has been contributing to the selection and expansion of resistant clones with new serotype/genotype combinations.

GBS causing invasive disease among neonates in Portugal are characterized by a limited number of serotypes and genetic lineages. While the dominance of serotype III and higher prominence of serotype Ia have been described worldwide, diversification within these serotypes appears to be ongoing, as illustrated by the increasing frequency of ST24 among serotype Ia and the recent emergence of highly resistant lineages within CC17. Antimicrobial resistance has a significant impact in clinical practice, not only in preventive strategies such as IAP but also in the empirical therapy for treatment of GBS infections. The increasing burden of GBS disease and dynamic nature of this pathogen substantiate the need for detailed characterization of the genetic lineages causing infections as well as for continued monitoring of antimicrobial resistance.

## Author contributions

EM, JM, and MR contributed to the work design; EM, CP, and PGSSI performed the acquisition and analysis of the data. EM, JM, and MR participated in the interpretation of the work, and co-wrote the paper. All authors read and approved the final paper.

## Funding

EM was supported by a grant from Fundação para a Ciência e a Tecnologia (SFRH/BPD/80038/2011). This work was partially funded by a grant from the governments of Iceland, Lichtenstein and Norway (EEA-PT06). The funders had no role in study design, data collection and interpretation, or the decision to submit the work for publication.

### Conflict of interest statement

JM has received research grants administered through his university and received honoraria for serving on the speakers bureaus of Pfizer, Bial, GlaxoSmithKline and Novartis. MR has received honoraria for serving on the speakers bureau of Pfizer and for consulting for GlaxoSmithKline. The other authors declare no conflict of interest. No company or financing body had any interference in the decision to publish.
