# Supplementary material for: Streptococcus agalactiae Causing Neonatal Infections in Portugal (2005–2015): Diversification and Emergence of a CC17/PI-2b Multidrug Resistant Sublineage
Source: Front Microbiol. 2017 Mar 28;8:499. doi: 10.3389/fmicb.2017.00499 (PMC5368217; doi:10.3389/fmicb.2017.00499)
Supplement: Supplementary file 1 [file Table1.pdf]

Supplementary Material

Streptococcus agalactiae causing neonatal infections in Portugal (2005-2015): diversification and emerging resistance within the hypervirulent CC17 lineage

Elisabete R. Martins, Cristiano Pedroso-Roussado, José Melo-Cristino, Mário Ramirez*, and the Portuguese Group for the Study of Streptococcal Infections

*** Correspondence:** Mário Ramirez: ramirez@medicina.ulisboa.pt

1 Supplementary Figures and Tables

Supplementary Table 1. Number of invasive neonatal disease cases and live births in Portugal by year

	No. per year										
	**2005**	**2006**	**2007**	**2008**	**2009**	**2010**	**2011**	**2012**	**2013**	**2014**	**2015**
Live births	109399	105449	102492	104594	99491	101381	96856	89841	82787	82367	85500
EOD[a]	17	13	8	16	12	8	9	5	7	9	9
LOD[a]	9	9	9	10	4	13	11	8	9	10	13
All	26	22	17	26	16	21	20	13	16	19	22

[a]EOD, early-onset disease; LOD, late-onset disease
